# Spontaneous Remission of Acute Myeloid Leukemia: A Case Report

**DOI:** 10.3390/medicina58070921

**Published:** 2022-07-11

**Authors:** Yolanda Martínez-Díez, Aida Franganillo-Suárez, Rocío Salgado-Sánchez, Mireia Atance-Pasarisas, Carlos Blas, María José Cotti-Ferrari, Tamara Castaño-Bonilla, Daniel Lainez-González, Socorro María Rodríguez-Pinilla, Pilar Llamas-Sillero, Juan Manuel Alonso-Dominguez

**Affiliations:** 1 Hematology Department, Fundación Jiménez Díaz University Hospital, ISS-FJD, 28040 Madrid, Spain; ymartinezd@salud.madrid.org (Y.M.-D.); aida.franganillo@quironsalud.es (A.F.-S.); rocio.salgado@quironsalud.es (R.S.-S.); mireia.atance@quironsalud.es (M.A.-P.); cblas@quironsalud.es (C.B.); mjose.cotti@quironsalud.es (M.J.C.-F.); tamara.castano@fjd.es (T.C.-B.); daniel.lainez@fjd.es (D.L.-G.); pllamas@fjd.es (P.L.-S.); 2 Pathology Department, Fundación Jiménez Díaz University Hospital, ISS-FJD, 28040 Madrid, Spain; smrodriguez@quironsalud.es

**Keywords:** leukemia, AML, spontaneous remission, molecular response

## Abstract

Spontaneous remissions (SRs) in acute myeloid leukemia (AML) are infrequent, poorly documented and transient. Similarly, morphological and cytogenetic complete remissions (CR) under azacitidine treatment are scarce. We report a 71-year-old man with a secondary AML arising from essential thrombocythemia (ET), who developed an SR after discontinuation of azacitidine following a respiratory infection (four courses were administered). The distinctive feature of our case is the depth of the achieved CR, documented by next-generation sequencing (NGS) techniques. We also detected persistence of molecular lesions that might already have been present in the previous ET clone. Our patient relapsed 5 months after achieving CR. We conclude that our patient showed a spontaneous remission of his AML rather than an exquisite response to azacitidine. We hypothesize that the concurrent respiratory infection, or any other unknown trigger, might have activated his immune system forcing the leukemic stem cell to enter a quiescent state through a yet unexplained mechanism.

## 1. Introduction

Acute myeloid leukemias (AML) arising from previous myeloproliferative neoplasms (MPN) are marked by a dismal prognosis [1]. Spontaneous remissions in AML are uncommon and not well-documented; the largest published series includes 46 patients [2]. SRs are often linked to infections or other stimulators of the immune system, suggesting that powerful immune activation plays a role in controlling the leukemic clone [2]. Nevertheless, the underlying mechanism of this phenomenon is not clearly understood.

Azacitidine therapy has shown an increase in overall survival (OS) compared to previous conventional care regimens [3]. However, CR was only detected in a small number of patients (19.5%), with an even more scarce rate of complete cytogenetic response (CCR). Namely, 2.1% of CCR were observed in the pivotal phase 3 trial that resulted in azacitidine approval for AMLs with >30% of blasts [4]. There is no data regarding molecular remissions under azacitidine treatment, but they might be scarcer than CCR rates.

Herein, we report a patient diagnosed with AML arising from essential thrombocythemia (ET), who developed disease remission once azacitidine treatment was discontinued while suffering a respiratory infection.

## 2. Case Description

We report a 71-year-old man with a medical history of ET with *JAK2^V617F^* mutation, diagnosed 10 years prior and treated with hydroxycarbamide, who presented to our department complaining of constitutional syndrome in November 2019. There were no relevant findings in the physical examination. Complete blood count (CBC) showed leukocytosis (16,000/mm^3^) with 27% of blasts, anemia (Hb 7.4 g/dL) and thrombocytopenia (69,000/mm^3^). Bone marrow aspirate was a dry tap so a biopsy was performed, showing massive blast infiltration, consistent with diagnosis of AML secondary to MPN.

Flow cytometry showed a 25% of cells CD45 + dim, CD34+, CD117+, HLA-DR+, CD13+, CD33+, CD71 + (10%), CD61 + (8%) and MPO + (16%). Cytogenetics found a complex karyotype: 46XY, del(5) (q13q33), der(7) t(7;) (p15; ?), del(9) (q22q32), del(13) (q14) [12]/46XY [1]. Fluorescence in situ hybridization (FISH) analysis revealed both 5q and 13q deletions in 82% and 78% of the analyzed nuclei, respectively. Neither *FLT3* (ITD or TKD) nor *NPM1* mutations were detected, yet an NGS panel revealed mutations in *JAK2^V617F^* (variant allele frequency (VAF): 60.9%), *TP53^R248Q^* (VAF: 60.9%) and *U2AF1^Y158_159dup^* (VAF: 42.5%) (Sophia Myeloid Solution, Sophia Genetics, Boston, MA, USA).

The patient underwent geriatric assessment and started hypomethylating therapy (azacitidine 75 mg/m^2^). Between the 3rd and 4th cycles he was admitted to hospital with an upper respiratory infection, requiring intravenous antibiotics for 5 days. Due to high transfusional requirements (18 red cell concentrates and 2 platelet pools), limited tolerability and absence of response, treatment was discontinued in April 2020, following the 4th cycle of azacitidine.

Since drug discontinuation, the patient’s CBC progressively improved, reaching peripheral blood (PB) counts similar to those observed previous to AML development (i.e., Hb 13 g/dL, 550,000/mm^3^ platelets and 9000/mm^3^ neutrophiles without immature cells). Bone marrow examination showed morphological CR (<5% of blasts in BM) with CCR (absence of abnormal metaphases in 20 metaphases analyzed) and negative minimal residual disease (MRD) by flow cytometry (<1 aberrant cell out of 1000 cells analyzed). NGS showed *JAK2* VAF: 44.3%, *U2AF1* VAF: 40.8%, and *TP53* VAF: 8.7% (NGS sensitivity: 3%). Monthly follow-ups as an outpatient were established, until in September 2020, cytopenias (Hb 8.1 g/dL, 73,000/mm^3^ platelets) and blasts in PB smear reappeared (11%). At this time, the patient received supportive care at home until his death in December 2020.

**Figure 1:**
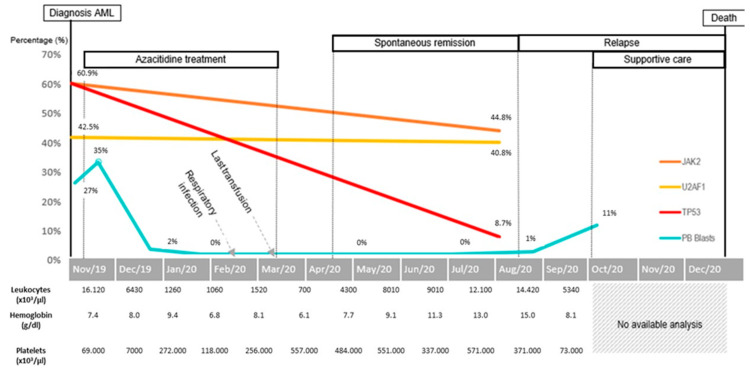
Timeline of complete blood count, peripheral blast count and molecular alterations detected in bone marrow.

The AML’s timeline from diagnosis to death is depicted in Figure 1. 

## 3. Methods

This report was a retrospective chart review. The patient had passed away at the time of publication. Fundación Jiménez Díaz Institutional Review Board approved submission of the article for publication in the journal.

## 4. Results and Discussion

Our patient had an AML arising from an MPN. It is well known that these kind of patients have an extremely dismal prognosis, even worse than in other secondary AMLs [1]. Disease remission was evidenced by morphologic, cytometric and cytogenetic techniques. AMLs secondary to MPN commonly show distinctive molecular and cytogenetic characteristics due to their different pathogenesis [5]. In particular, mutations of *TP53*, *TET2*, *ASXL1*, *IDH1*/*2*, *U2AF1*, *SRSF2* and *RAS* family genes suggest leukemic evolution in MPN [6,7,8]. In our patient we detected *TP53^R248Q^* and *U2AF1^Y158_159dup^* mutations at time of leukemic transformation. NGS showed a marked reduction in *TP53* VAF, while only a minimal reduction was seen in the *JAK2* and *U2AF1* clones. This finding was consistent with documented AML remission with persistence of mutations that might be already present in the ET clone. Regrettably we had no sample available from the ET phase of the patient.

To our knowledge, there are only 3 SR reported cases with a complex karyotype and we report the first case of *TP53* mutant AML achieving SR [2,9]. Additionally, as far as we know this is the first reported SR which includes molecular data from NGS studies. Interestingly, the number of SRs with high-risk cytogenetics is comparable to that in the overall diagnosis of AML [2]. Therefore, cytogenetics might not be relevant in achieving SRs.

Azacitidine implementation in AML treatment has shown that achievement of CR is not mandatory to improve survival of AML patients [10]. In fact, as previously explained, CR are scarce and CCR are even less common [4]. Additionally, these responses are achieved under treatment that is normally administered until disease progression. Our patient only received four courses of azacitidine before withdrawal of treatment due to clinical worsening. After discontinuation, remission was unexpectedly achieved, although after two cycles of azacitidine a PB smear had already shown no blasts, in spite of having 27% of PB blasts at diagnosis. Altogether, these findings suggest that our patient had a SR instead of an exquisite response to azacitidine.

SRs in AML are an uncommon phenomenon, rarely well documented. In total, 90% of reported SRs achieve CRs [2]. Response is commonly maintained for a short period of time—a mean of 5 months, like in our case. SRs have been associated with certain stimulators of the immune system, such as infections [2]. However, in some SRs no trigger was identified [11]. Our patient suffered from pneumonia, though no microorganism was identified in blood cultures, and we believe this could be the cause of the SR. Nevertheless, a drastic decrease of PB blasts had already been observed previous to the infection. This finding is not compatible with an infectious origin of the SR, and so we must be very cautious with our approach. Still, most of the published SRs presented with pneumonias or respiratory symptoms of bacterial etiology [12,13]. Moreover, some studies suggest that polyclonal hypergammaglobulinemia, TNF-α and IL-2 levels may take part in this response [14,15]. Despite reports on the possible anti-leukemic effect of transfused leukocytes (inducing a kind of graft-versus-leukemia activity), nowadays this seems unlikely due to routine use of leukocyte depleted blood components in patients with AML [9,16]. Other potential trigger factors described are corticosteroids or granulocyte colony stimulating factor (G-CSF) administration [17]. Interestingly, *NPM1* mutation has been suggested to facilitate SR in AML due to a greater sensibility to oxidative stress [18].

At the present time, the underlying mechanism of SRs is unclear. However, we could hypothesize that somehow the infection, or any other previous unknown trigger, induced immune activation against AML [2]. A possible explanation could be that the immune system forces the leukemic stem cell (LSC) to enter a quiescence state through an unknown mechanism. Cytokines produced as a result of the unknown trigger could either act directly on the LSC or through the bone marrow microenvironment and stop LSC replication. This lack of replication extinguishes the progeny of LSC. Whatever the mechanism is, it stops the LSC from re-entering the cell cycle and thus abrogates AML disease expression. Shedding light on this unexplained mechanism could help us decipher LSC quiescence regulation and possibly open new therapeutic paths. Additionally, the plausible immune origin of SR highlights the importance of immune targeting AML.

## 5. Conclusions

SR is an uncommon and transient phenomenon thought to be the consequence of different triggers that might force the LSC to enter in a quiescent state. A prospective study of these cases, measuring the cell cycle of the LSC and cytokines of the patient, could help us to decipher the mechanism of regulation of the quiescence of LSC which could have interesting therapeutic implications.

## Data Availability

The datasets used and/or analyzed during the current study are available from the corresponding author on reasonable request.
